# Sudden Cardiac Death in a Patient with Anomalous Origin of Right Coronary Artery with Concomitant Sinus of Valsalva Aneurysm

**DOI:** 10.7759/cureus.7377

**Published:** 2020-03-23

**Authors:** Adnan Ahmed, Mariam Mir, Sarthak Soin, Sabah Patel

**Affiliations:** 1 Internal Medicine, Saint Joseph Hospital, Chicago, USA; 2 Internal Medicine, Presence Saint Joseph Hospital, Chicago, USA; 3 Internal Medicine, Rosalind Franklin University of Medicine and Science, Chicago, USA

**Keywords:** anomalous rca, sova, sudden cardiac death, coronary artery, chest pain

## Abstract

Anomalous origin of right coronary artery (ARCA) arising from the left sinus of Valsalva (a congenital cardiac abnormality) and sinus of Valsalva aneurysm (SOVA) are rare and often have asymptomatic conditions. However, symptoms could range from chest pain, shortness of breath, palpitations, syncope to sudden cardiac death (SCD). The co-existence of these two anomalies (ARCA + SOVA) could lead to potential adverse outcomes in the absence of early intervention. The presence of these two conditions together increases the risk of SCD as reported in our case of a young male who presented to the emergency department with chest pain.

## Introduction

Anomalous origin of right coronary artery (ARCA) from the left sinus of Valsalva and its malignant course is a rare congenital anomaly; however, its association with aneurysm of left sinus of Valsalva is a rare co-existence. The angiographic incidence of the anomalous origin of the coronary artery is approximately 0.2% to 1.3%. The incidence of ARCA is noted to be 0.107% and was first described in 1948 by White and Edwards [1,2]. Sinus of Valsalva aneurysm (SOVA) is an abnormal dilatation of the aortic root found between the sino-tubular junction and aortic annulus. It can be congenital as well as acquired [3]. The incidence of SOVA is 0.09% based on autopsy series and constitutes 0.1% to 3.5% of all congenital heart disease. It has a marked male predominance and has the highest incidence in Asian populations [4,5]. These findings can remain asymptomatic for years and are often discovered incidentally.

## Case presentation

A 45-year-old male with a past medical history of human immunodeficiency virus (HIV) on highly active anti-retroviral therapy (HAART) presented to the emergency department with complaints of retrosternal chest pain and dark stool for one week. On admission, his vitals were blood pressure 136/74 mmHg, heart rate 89 beats/min, respiratory rate 16/min, oxygen saturation 96% on room air, and temperature 37.7 °C. Physical examination was positive for dark stool in the rectal vault which tested positive on bedside fecal occult blood test (hemoccult).

**Figure 1:**
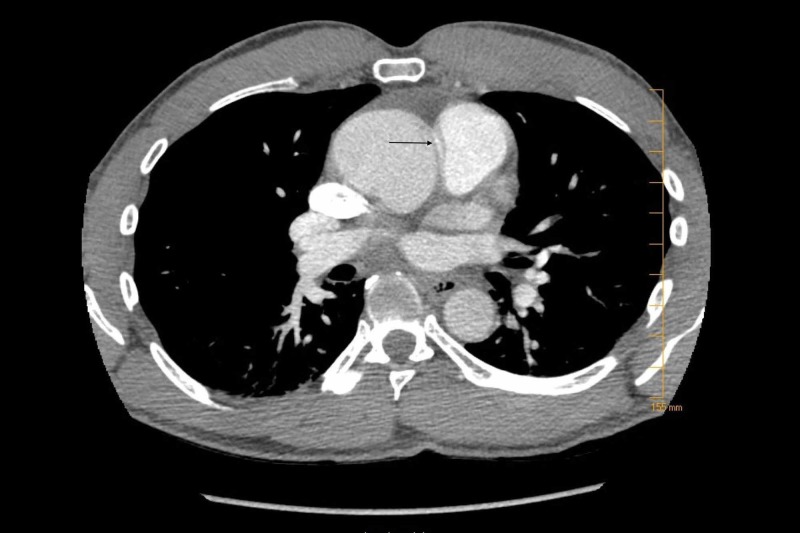
Computed tomography (CT) of the chest (axial view); arrow showing aberrant origin right coronary artery

**Figure 2:**
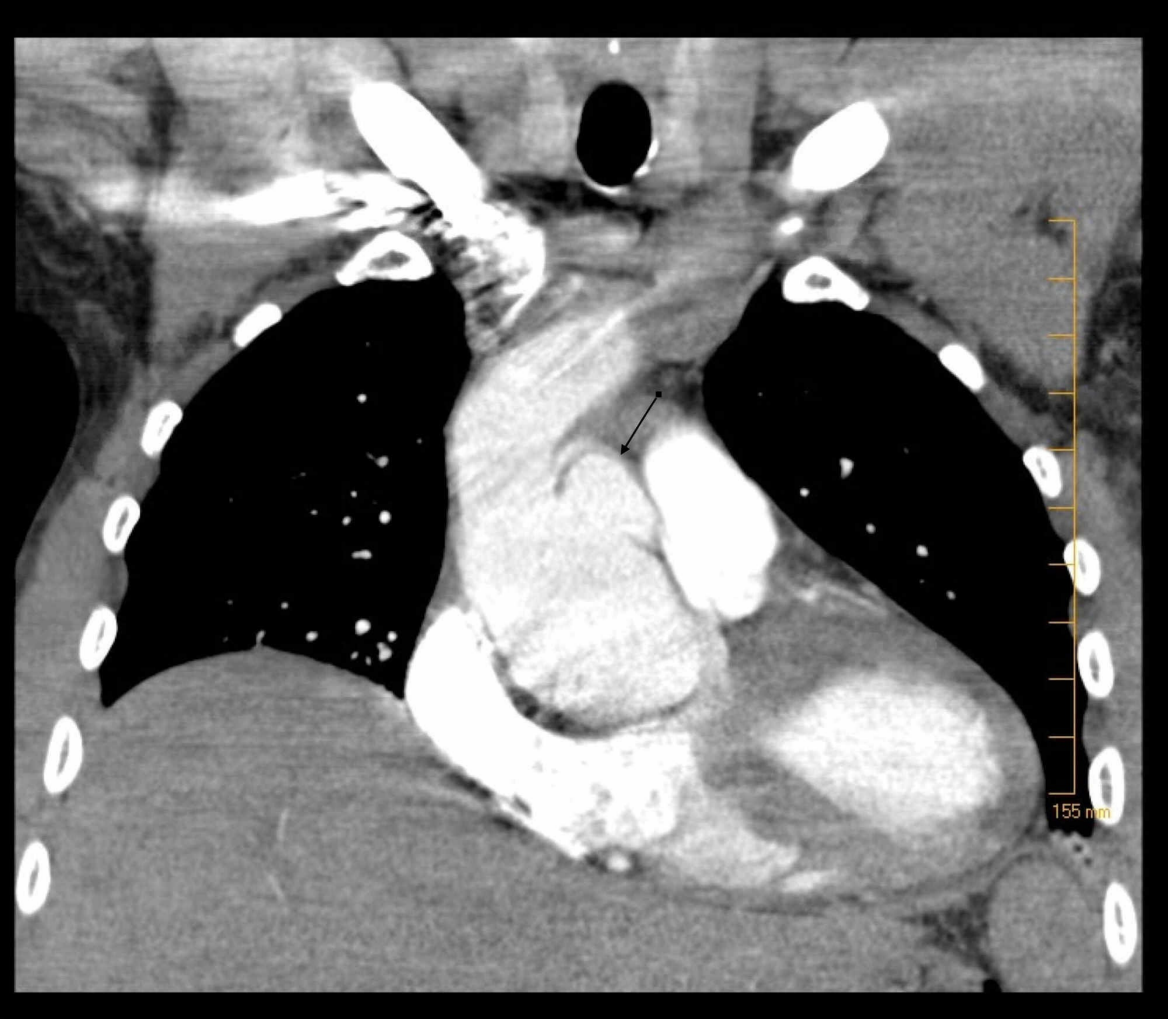
Computed tomography (CT) of the chest (coronal view); arrow showing 3 x 2 cm aneurysmal dilatation of the left sinus of Valsalva

Labs were remarkable for troponin <0.03 ng/m, serum creatinine 1.55 mg/dl, potassium 5 meq/l, bicarbonate 32 meq/l, hemoglobin 15.5 g/l and (international normalized ratio (INR) 1.1, and D-dimer of 2800 (normal < 500 ng/L). An electrocardiogram (EKG) showed normal sinus rhythm with right bundle branch block. Computed tomography (CT) of the chest with contrast was performed to rule out pulmonary embolism which in turn showed aberrant origin of the right coronary artery from the left sinus of Valsalva, demonstrating a malignant course between the aorta and the pulmonary artery. Additionally, there was aneurysmal dilatation of the left sinus of Valsalva measuring 3 x 2 cm (Figures 1-2). Cardiology, cardiothoracic surgery, and gastroenterology was consulted. He was admitted for cardiac monitoring and an hour after being transferred to the medical ward was found unresponsive. Cardiac monitor showed asystole and cardiopulmonary resuscitation was initiated per advanced cardiac life support (ACLS) protocol. Return of spontaneous circulation was achieved in 30 minutes and the patient was transferred to the intensive care unit (ICU). A second episode of asystole occurred on arrival to the ICU from which the patient could not be revived and led to his demise.

## Discussion

Since its first description in 1948, ARCA is now known to be the second leading cause of sudden cardiac death (SCD) after hypertrophic cardiomyopathy in young athletes. Pathophysiology, although poorly understood, is presumed to be mechanical compression of RCA by great vessels especially during periods of exercise and stress which over time leads to coronary ischemia [6].

SOVA as a distinct entity is associated with increased morbidity and mortality. Aneurysms may originate in the right coronary sinus (most common site 65%-85%), the noncoronary sinus (10%-30%), and uncommonly the left coronary sinus (1%-5%). A deficiency of normal elastic tissue and abnormal development of the bulbus cordis have been associated with the development of SOVA. Other disease processes that involve the aortic root (e.g., atherosclerotic aneurysms, syphilis, endocarditis, cystic medial necrosis, chest trauma) are also well-known causes of acquired aneurysms. Approximately 25% of reported cases of SOVA are clinically asymptomatic [7]. Our patient’s syphilis status was known to be negative.

The co-existence of both of these anomalies in the same patient is very rare and would likely be the least suspected cause of chest pain in a given differential. Both conditions can present with chest pain, shortness of breath, arrythmias, syncope, and SCD. Only 20% of coronary anomalies produce life-threatening symptoms [8]. Initial cardiac testing like EKG and troponin may be unrevealing. They are usually detected on imaging like CT chest, magnetic resonance imaging (MRI), coronary angiograms or transesophageal echocardiogram. Early diagnosis and prompt treatment are necessary to prevent SCD [5].

Both conditions may require emergent surgical correction vs. elective surgery based on their clinical presentation. Previously, medical therapy and/or percutaneous stenting and surgery were all considered as appropriate options for the treatment of ARCA, but now we see a change in treatment strategy favoring surgical treatment in symptomatic patients [9].

Ruptured sinus of Valsalva requires surgical management, but endovascular devices have shown good outcomes. Regardless of the intervention, urgent cardiothoracic surgery evaluation is the standard of care. Non-ruptured aneurysm may be monitored but should be urgently repaired if there are significant changes in symptoms or show rapid enlargement. The guidelines from 2010 Thoracic Aortic Disease recommend surgical repair with aneurysms greater than 5.5 cm and 5 cm in those with bicuspid valves and 4.5 cm in the setting of connective tissue disease. Repair should also be considered when there is a growth rate of more than 0.5 cm/year [10,11].

## Conclusions

The co-existence of ARCA from the left sinus of Valsalva and SOVA is very rare but should be considered in the differential diagnosis for all patients presenting with chest pain. The presence of these conditions together increases the risk of adverse outcomes and SCD. Early diagnosis and intervention are the only means to prevent mortality. They are usually detected incidentally on imaging like CT chest, coronary angiograms, or transesophageal echocardiography. However, once detected, patients should be monitored in the ICU until evaluation for definitive management can be completed.
